# Cross-Modal Weakly Supervised RGB-D Salient Object Detection with a Focus on Filamentary Structures

**DOI:** 10.3390/s25102990

**Published:** 2025-05-09

**Authors:** Yifan Ding, Weiwei Chen, Guomin Zhang, Zhaoming Feng, Xuan Li

**Affiliations:** School of Electrical and Information Engineering, Wuhan Institute of Technology, Wuhan 430205, China; 15239482556@163.com (Y.D.); roger_0122@163.com (W.C.); 15234327504@163.com (G.Z.);

**Keywords:** weak supervision, saliency object detection (SOD), multi-source annotations, RGB-D

## Abstract

Current weakly supervised salient object detection (SOD) methods for RGB-D images mostly rely on image-level labels and sparse annotations, which makes it difficult to completely contour object boundaries in complex scenes, especially when detecting objects with filamentary structures. To address the aforementioned issues, we propose a novel cross-modal weakly supervised SOD framework. The framework can adequately exploit the advantages of cross-modal weak labels to generate high-quality pseudo-labels, and it can fully couple the multi-scale features of RGB and depth images for precise saliency prediction. The framework mainly consists of a cross-modal pseudo-label generation network (CPGN) and an asymmetric salient-region prediction network (ASPN). Among them, the CPGN is proposed to sufficiently leverage the precise pixel-level guidance provided by point labels and the enhanced semantic supervision provided by text labels to generate high-quality pseudo-labels, which are used to supervise the subsequent training of the ASPN. To better capture the contextual information and geometric features from RGB and depth images, the ASPN, an asymmetrically progressive network, is proposed to gradually extract multi-scale features from RGB and depth images by using the Swin-Transformer and CNN encoders, respectively. This significantly enhances the model’s ability to perceive detailed structures. Additionally, an edge constraint module (ECM) is designed to sharpen the edges of the predicted salient regions. The experimental results demonstrate that the method shows better performance in depicting salient objects, especially the filamentary structures, than other weakly supervised SOD methods.

## 1. Introduction

Salient object detection (SOD) aims to emulate human visual attention by capturing the most salient objects and regions in an image, which offers crucial cues for higher-level visual tasks such as image retrieval [1,2,3], object tracking [4], image quality assessment [5], scene analysis [6,7], object classification [8], and autonomous driving [9]. Fully supervised SOD tasks require extensive pixel-level annotation, which is time-consuming and labor-intensive [10,11,12,13,14]. The emergence of weakly supervised learning methods effectively addresses the data labeling issue and enhances the transferability of the model. However, weakly supervised methods [15,16,17,18,19] usually rely on image-level labels or limited annotation information, and it is difficult for weakly supervised methods to provide sufficient annotation information as guidance during the model’s training.

To enrich the information of annotation, researchers propose multi-source weakly supervised methods. However, obtaining high-quality pseudo-labels is still difficult. Low-quality pseudo-labels are inadequate in enabling the model to recognize complete edges, particularly filamentary structures. Since semantic information provides feature representations of the object and visual information provides its positional information, this work selects complementary cross-modal weak labels to generate supervisory information that is both rich and accurate. Moreover, as depth images provide rich geometric information [20,21,22,23,24], an increasing number of studies have utilized depth images as a crucial 3D spatial cue for SOD tasks. Depth images help the model distinguish salient objects from the background by perceiving the depth structure of the scene, which is particularly useful in complex scenes. However, there are significant differences among different modalities. For example, RGB images contain rich contextual information, while depth images primarily provide geometric information. This difference makes it difficult for traditional fusion methods such as channel concatenation or element-wise summation to effectively utilize the complementary information of the two modalities. Therefore, when fusing different modalities [8,25,26,27], researchers must design complex network structures and loss functions. At the same time, the number of existing RGB-D datasets is limited, and some datasets have low annotation quality, which results in the inability to meet the training requirements of models. Therefore, in the context of weakly supervised learning, it remains a challenge to utilize multi-modal and multi-dimensional data to address the issue of missing salient regions in complex scenarios, especially the detection of filamentary structures.

To address the aforementioned issue, this approach explores the complementarity of cross-modal weak labels by combining the semantic information of text labels with the visual information of point labels, thereby enriching the dimensionality of the supervisory information. Meanwhile, it enhances the utilization of the advantageous properties of RGB and depth images, providing the saliency detection model with a more comprehensive understanding of the scene. Specifically, a cross-modal pseudo-label generation network (CPGN) is designed, which leverages the semantic information contained in text labels as prior knowledge and integrates the powerful image–text matching capability of CLIP to generate initial pseudo-labels. The annotations and positional information provided by point labels are then employed to hierarchically refine the initial pseudo-labels, providing high-quality pseudo-labels aligned with human perception for salient object detection. To fully leverage the contextual and geometric features, an asymmetric salient-region prediction network (ASPN) is proposed to capture the advantageous features of different images. Various attention mechanisms are embedded to explore the correlation between RGB and depth features, enabling efficient coupling of multi-dimensional information. Additionally, an edge constraint module (ECM) is integrated into the ASPN to sharpen the edges of the saliency results. By efficiently combining multi-modal and multi-dimensional information, the proposed approach achieves salient object detection in complex backgrounds under a weakly supervised learning framework and demonstrates outstanding performance in filamentary structure detection.

The main contributions of this study are as follows:

1. We propose a cross-modal weakly supervised method for the RGB-D SOD task, in which the text and point annotations are used to provide rich semantic information and position information, respectively. By fully integrating the advantages of weak labels from different modalities, the method effectively enhances the detection performance of salient objects in complex scenes, especially in highlighting filamentary structures.
2. A cross-modal pseudo-label generation network (CPGN) is designed to provide stronger supervision for model training, which first leverages the multimodal alignment capability of CLIP to activate semantic priors from text labels, obtaining coarse annotations, and then point labels progressively enhance the richness and accuracy of these annotations to generate high-quality pseudo-labels.
3. An asymmetric salient-region prediction network (ASPN) is proposed to extract and integrate the contextual information of RGB images and the geometric information of depth images. In ASPN, an edge constraint module is introduced to sharpen the edges of salient objects. Meanwhile, we construct a cross-modal weakly supervised saliency detection dataset (CWS) for the weakly supervised RGB-D SOD task.

## 2. Related Work

### 2.1. Weakly Supervised Salient Object Detection

Salient object detection (SOD) has garnered increasing attention, and although current methods have achieved excellent results, they rely on time-consuming and labor-intensive pixel-level manual annotations. As a result, weakly supervised strategies have become mainstream. These strategies use image-level labels [18,28,29], scribbles [30,31,32], bounding boxes [33], and points [34] to supervise model training. For example, Li et al. [28] and Yu et al. [31] perform self-training by using predicted saliency maps as part of the pseudo-labels. Zeng et al. [35] employs multi-task learning, including multi-label classification and caption generation, to assist in generating pseudo-labels. Liu et al. [33] iteratively refine pseudo-labels by learning a multi-task mapping refinement network with salient bounding boxes. Point-based methods, such as the PSOD proposed by Piao et al. [34], integrate point labels and edges to refine pseudo-labels.

Our research focuses on leveraging the powerful capabilities of CLIP, using it to convert image–text similarity into pixel-text matching for precise segmentation, while also utilizing the strong foreground–background differentiation ability of point labels to create complete pseudo-labels.

### 2.2. Weakly Supervised RGB-D Salient Object Detection

With the advancement of depth imaging technology, depth maps have been introduced as a crucial 3D cue in the SOD task. Depth maps enhance the ability of SOD models to detect salient objects in complex scenes, leading to the rapid development of RGB-D salient object detection. Meanwhile, in order to reduce the cost of pixel-level annotations, weakly supervised RGB-D SOD solutions need to be further explored. Xu et al. [17] addressed the sparsity issue of scribbles through a secondary labeling method. Li et al. [15] modeled the pixels in RGB images to supplement the missing pixel-level annotations in scribbles.

The method leverages the attribute advantages of cross-modal weak labels. Meanwhile, it utilizes the Swin-Transformer and a CNN network to create a dual-branch architecture for feature extraction, and it proposes an edge constraint module to refine the edge details of the salient object.

### 2.3. Contrastive Language–Image Pre-Training

CLIP (Contrastive Language–Image Pre-Training) is a visual–language pre-training model introduced by OpenAI [36,37]. Its core idea is to establish a correlation between images and text, enabling better mutual understanding through measuring the similarity between images and text. CLIP is implemented by combining and matching a large number of images with their corresponding textual information to build a large-scale dataset. CLIP can pair input images with text and compute similarity scores between them. In practice, for an input image or text, the model outputs similar images or text, facilitating more intelligent information processing.

Most existing weakly supervised SOD methods use CAMs as pseudo-labels. However, standard CAMs are often noisy, which can significantly degrade model performance. This method uses CLIP to generate class activation maps (CAMs), delving into the relationship between text and objects in images to create a robust foundation for obtaining results that meet our requirements.

## 3. Proposed Method

### 3.1. Overview

**Figure 1:**
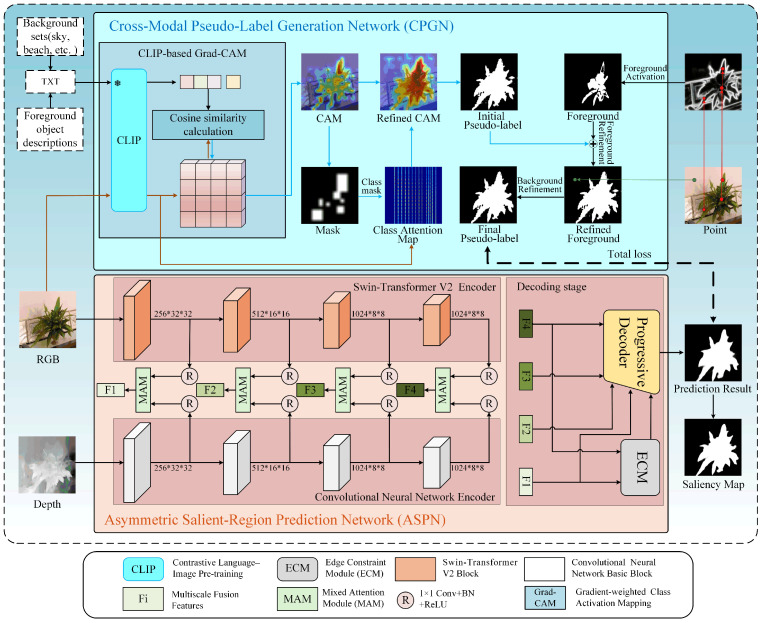
The overall architecture of the proposed model. A CPGN is used for generating high-quality pseudo-labels, while an ASPN is used for salient object detection.

We solve some existing issues in the weakly supervised salient object detection (SOD) task for RGB-D images, e.g., the insufficient supervision ability of weak labels, the incomplete prediction of object regions and the failure to detect filamentary structures. The overall framework we proposed is shown in Figure 1. Among them, it can be divided into a cross-modal pseudo-label generation network (CPGN) and an asymmetric salient-region prediction network (ASPN). Firstly, we designed the CPGN, which takes as input $D={\left\{R_{i},T_{i},P_{i}\right\}}_{i=1,2,3,\ldots ,n}$, where *i* is the index of dataset samples and $\left\{R_{i},T_{i},P_{i}\right\}$ represents the paired RGB image ($R_{i}$), text labels ($T_{i}$), and point labels ($P_{i}$). The network outputs high-quality pseudo-labels ($P^{h}$) to guide the ASPN to predict results with complete regions, especially for the detection of filamentary structures. Secondly, in view of the attribute differences between RGB images and depth images, the ASPN employs an asymmetric encoder structure based on the Swin-Transformer and a CNN to progressively extract the features of RGB-D images. The structure is designed to enable the ASPN to fully extract contextual information from RGB images while making the ASPN more sensitive to the spatial structure information in depth maps. Finally, we designed an edge constraint module (ECM), which utilizes shallow features and deep features to generate edge features to sharpen the edges of the salient objects by the ASPN. In conclusion, this method capitalizes on the synergy of the two networks to tackle the issues of insufficient supervision of weak labels, incomplete salient regions, and the absence of filamentous structures in current weakly supervised SOD tasks for RGB-D images.

### 3.2. Cross-Modal Pseudo-Label Generation Network

#### 3.2.1. CLIP-Based Grad-CAM

Class activation mapping (CAM) has been widely used in weakly supervised SOD tasks. However, the pseudo-labels generated only by using CAM often contain noise, and the separation between the foreground and the background is not satisfactory, which makes it difficult to directly use them to guide the model training. To achieve better results, we divide the text label activation task into two stages: pseudo-label generation and refinement.

The text labels are obtained by calibrating the Caps dataset in [38]. The text labels consist of 122 foreground object descriptions (such as cat, dog, and people riding on bicycles) and 23 common background classes (such as sky, ground, and beach). As shown in Figure 1, the RGB image and the text labels (foreground label and background set) of the image are fed into the CPGN simultaneously.

We need to match the information from the text labels with the object regions in the image, and the CLIP model is well suited for this task due to its strong zero-shot capability. Therefore, our text labels include both foreground information of the object and a common background set. We use the softmax function to ensure mutual exclusivity between different classes, thereby suppressing the background and achieving good segmentation performance in the presentation of the matching results. Specifically, the scores after applying softmax are calculated as follows:

(1) $$s^{c}=\frac{exp\left(y^{c}\right)}{\sum_{c^{\prime }=1}^{C}exp\left(y^{c^{\prime }}\right)}$$

where $s^{c}$ represents the score for the c-th object text, *C* is the total number of object texts, and $y^{c}$ is the cosine similarity score for the c-th object.

We use Grad-CAM as the visualization method for the matching results. Grad-CAM is a visualization tool for deep learning models that allows us to intuitively understand the regions of the image that CLIP focuses on when given an input object text. It then visualizes this result and generates CAMs $M_{c}$. The specific formula is as follows:

(2) $$\begin{array}{c} \alpha_{k}^{c}=\frac{1}{Z}\sum_{i}\;\sum_{j}\;\frac{\partial y^{c}}{\partial A_{ij}^{k}}*s^{c}\left(1-s^{c}\right)\\ \begin{array}{cc} & +\frac{1}{Z}\sum_{i}\;\sum_{j}\;\sum_{c^{\prime }\neq c}\;\frac{\partial y^{c^{\prime }}}{\partial A_{ij}^{k}}*s^{c}\left(-s^{c^{\prime }}\right) \end{array} \end{array}$$

(3) $$M_{c}=ReLU\left(\sum_{k}\;\alpha_{k}^{c}A_{ij}^{k}\right)$$

where $\alpha_{k}^{c}$ is the weight of the k-th channel in the last feature layer A of the CLIP model for the c-th class, *Z* is the total number of pixels, $A_{\mathrm{ij}}^{k}$ is the pixel coordinate $\left(i,j\right)$ in the k-th channel of the last feature layer A of the CLIP model, and $\frac{\partial y^{c}}{\partial A_{\mathrm{ij}}^{k}}$ is the gradient information of class *c* backpropagated through the k-th channel of the last feature layer A in the CLIP model.

Finally, the result refinement is achieved by combining the CAMs $M_{c}$ of each class *c* with the attention weights $W_{a}$ from the CLIP image encoder. Specifically, to make $W_{a}$ pay more attention to the object, we threshold the CAMs to obtain masks and then cover the regions related to category *c* in the masks with boxes to obtain class masks $B_{c}$. After that, we perform sinkhorn normalization and transpose sum processing on $W_{a}$, respectively, to enhance the object weights. By multiplying $B_{c}$ by $W_{a}$, we obtain the class attention map. We use the semantic similarity among pixels in the class attention map to refine the CAM, thereby covering more incomplete areas of the object in $M_{c}$. The generation of our initial pseudo-labels $M^{\mathrm{init}}$ is shown in the following equation:

(4) $$M^{init}=B_{c}⊙\frac{W_{A}+{\left(W_{A}\right)}^{T}}{2}\cdot vec\left(M_{c}\right)$$

(5) $$W_{A}=Sinkhorn\left(W_{a}\right)$$

where $B_{c}$ is the class mask, Sinkhorn(·) is the sinkhorn normalization, and $vec\left(\cdot \right)$ denotes matrix vectorization. Since the class activation map is essentially the confidence of each pixel in the image regarding class *c*, a hyperparameter λ (i.e., the filtering parameter) is set to ignore pixels with low confidence, completing the text label activation process, as represented by:

(6) $$P^{init}=\left\{\begin{array}{c} 1,M_{\left(i,j\right)}^{init}>\lambda \\ 0,M_{\left(i,j\right)}^{init}<\lambda \end{array}\right.$$

where $P^{\mathrm{init}}$ is the initial pseudo-label and $\left(i,j\right)$ represents the coordinates of a pixel in the class activation map $M^{\mathrm{init}}$.

#### 3.2.2. Point Label Activation and Pseudo-Label Improvement

Due to the semantic ambiguity of text labels, the masks generated from them contain a lot of noise, which causes the model to learn incorrect information. To address this issue, we introduce point labels into the pseudo-label generation process. The method leverages the advantages of multiple modalities, refining the initial pseudo-labels from both the foreground and background to obtain the final pseudo-label with sufficient supervisory information. We fully leverage the advantages of point labels to improve the quality of pseudo-labels. We divide point labels into foreground points $S^{f}$ and background points $S^{b}$. The flood fill algorithm is a method used to fill a connected region with a specified color or value, starting from a given seed point. Using the flood fill algorithm, we fill the edge E detected by the edge detector [39] with the foreground point labels to obtain the foreground activation map $P^{p}$. In this method, the foreground point is set as the seed point, with a radius of one-fifth of the given image size, and the Low Difference and High Difference are set to 20 and 50, respectively. The specific operation is defined as:

(7) $$P^{p}=FF\left(S^{f},e\right)$$

where $FF\left(\cdot \right)$ represents the flood fill algorithm. Then, we add the foreground activation map pixel-wise with the initial pseudo-labels to complete the foreground refinement process, followed by using the background points as seed points to flood fill the entire image to remove non-object noise and obtain the final pseudo-label $P^{h}$. The specific operation is defined as:

(8) $$P^{h}=FF(S^{f}-S^{b},P^{init}⊕P^{p})$$

We use $P^{h}$ to guide the training of the ASPN.

### 3.3. Asymmetric Salient-Region Prediction Network

#### 3.3.1. Asymmetric Encoder

Weakly supervised SOD tasks for RGB-D images typically employ encoder structures based on ResNet, VGG, or Transformer backbones. These fundamental encoders are commonly pre-trained on RGB images to initialize the model. However, recent studies have found attribute differences between RGB and depth images. The extraction of depth image features using these encoders may introduce interference in the network. Depth images contain rich spatial positional information, and CNN-based encoders are more sensitive to spatial structures. Therefore, we use CNN-based encoders to extract features from depth images. Moreover, encoders based on the Swin-Transformer are better equipped to capture contextual information. Therefore, we use the Swin-Transformer encoder to extract features from RGB images.

We extract hierarchical features from the RGB features. Specifically, the Swin-Transformer first divides the input RGB image I into non-overlapping P×P patches, which are then flattened and represented as:

(9) $$X=Linear\left(Patch\left(I\right)\right)$$

**Figure 2:**
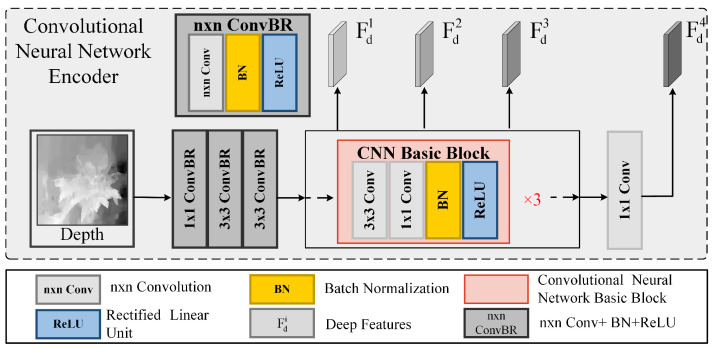
The architecture of the CNN-based encoder. It is used to progressively extract features from depth images.

Here, $Patch\left(\cdot \right)$ refers to dividing the RGB image into P×P patches, and $Linear\left(\cdot \right)$ is the operation that transforms each patch into a higher-dimensional embedding feature space. The Swin-Transformer updates features layer by layer through hierarchical feature extraction. Each layer l consists of two core operations:

(a) Performing multi-head self-attention operations on each patch:
  (10) $$Z^{l}=W-MSA\left(X^{l}\right)+X^{l}$$
  Here, $W-MSA\left(\cdot \right)$ refers to the window-based multi-head self-attention operation, $X^{l}$ is the input feature at layer l, and $Z^{l}$ is the feature after the window-based multi-head self-attention operation.
(b) To better capture the contextual relationships, multi-head self-attention operations are performed between windows:
  (11) $$X^{l+1}=SW-MSA\left(Z^{l}\right)+Z^{l}$$
  Here, $SW-MSA\left(\cdot \right)$ refers to the cross-window multi-head self-attention operation. By stacking multiple layers of such operations, contextual features are gradually extracted. The final output is the contextual features $\left\{F_{\mathrm{rgb}}^{i}|i=1,2,3,4\right\}$ after hierarchical feature extraction. Depth features are typically used to supplement the spatial positional information of the RGB features. Networks trained from scratch tend to achieve better performance in tasks related to depth maps. Therefore, we adopted a CNN-based backbone to extract depth features in the simplest way possible. As shown in Figure 2, the depth map is converted into a higher-dimensional embedding feature space through convolution layers with different receptive fields. Then, the learned depth features are transformed into multi-scale features with three different resolutions via three identical CNN-based blocks.

Finally, for the fourth stage, we use a 1 × 1 convolution layer to obtain the final output. In this way, we acquire the depth features, represented as $\left\{F_{d}^{i}|i=1,2,3,4\right\}$. We use a serial structure in the depth branch, which is capable of extracting deeper information and helps reduce structural distortion during the feature extraction process of depth maps.

#### 3.3.2. Mixed Attention Module (MAM)

**Figure 3:**
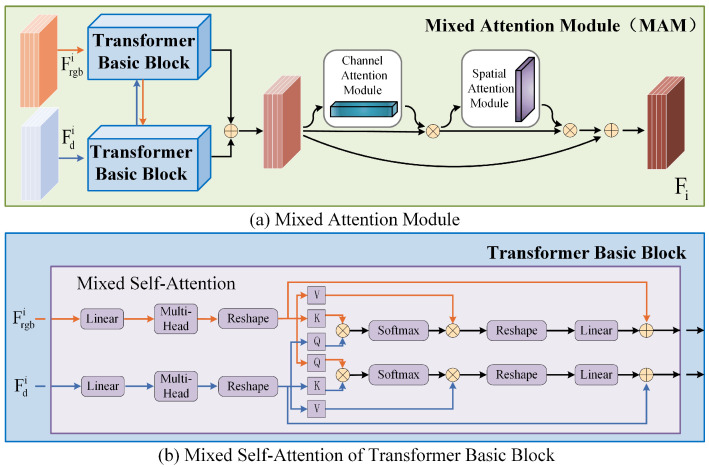
The architecture of the mixed attention module.

As shown in Figure 3, we feed the RGB features $\left\{F_{\mathrm{rgb}}^{i}|i=1,2,3,4\right\}$ and the depth features $\left\{F_{d}^{i}|i=1,2,3,4\right\}$ extracted from the asymmetric encoder into the Transformer basic blocks. The cross-modal information is output by mixing their respective attention weights in the mixed self-attention layer. Specifically, before the mixed self-attention layer, we extract the query Q, key K, and value V from both $F_{\mathrm{rgb}}^{i}$ and $F_{d}^{i}$. as $Q_{R},\;K_{R},\;V_{R}$ and $Q_{D},\;K_{D},\;V_{D}$, respectively. We use $Q_{R}$ along with $K_{D}$ and $V_{D}$ as the head in the RGB feature stream, while $Q_{D}$ is used together with $K_{R}$ and $V_{R}$ as the head in the depth feature stream. This process is defined as:

(12) $$Head_{rgb}=MHSA\left(Q_{R},\;K_{D},\;V_{D}\right)$$

(13) $$Head_{d}=MHSA\left(Q_{D},\;K_{R},\;V_{R}\right)$$

After mixing the two through mixed self-attention, the output information is summed to obtain the attention weights:

(14) $$MHSA_{rgb}=Cat\left(\sum_{n=1}^{n}Head_{rgb}^{j}\right)W_{1}+F_{RGB}$$

(15) $$MHSA_{d}=Cat\left(\sum_{n=1}^{n}Head_{d}^{j}\right)W_{1}+F_{D}$$

where $W_{1}$ and $W_{2}$ are linear projections. After obtaining the feature maps $\{F_{j}^{i}|i=$1,2,3,4,j=rgb,d} mixed through the Transformer basic blocks, we further utilize spatial attention and channel attention to better fuse the features of different modalities. This helps generate the final multi-scale features at each scale, $\left\{F_{i}|i=1,2,3,4\right\}$, with the following operations:

(16) $$F_{i}=F_{j}^{i}⊕\left(F_{j}^{i}⊗\mathrm{SA}\left(F_{j}^{i}⊙\mathrm{CA}\left(F_{j}^{i}\right)\right)\right)$$

Here, $\mathrm{CA}\left(\cdot \right)$ and $\mathrm{SA}\left(\cdot \right)$ represent channel attention and spatial attention, respectively.

#### 3.3.3. Edge Constraint Module (ECM)

**Figure 4:**
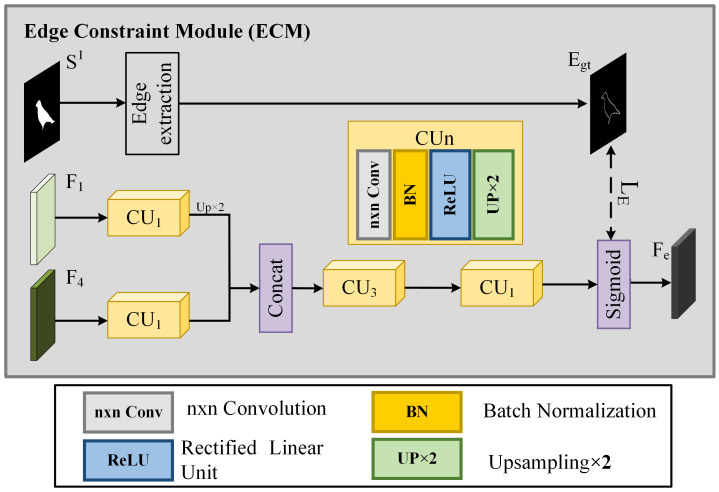
The architecture of the edge constraint module. It provides edge information for the ASPN.

Complete edges can enhance the performance of saliency detection models. Therefore, we propose an edge constraint module (ECM), which progressively refines the output of edge features. Low-level features contain rich texture information, while high-level features contain rich semantic information. The ECM combines multi-scale features to sharpen the edges of the salient object. Figure 4 illustrates the schematic diagram of our edge constraint module. The module is divided into two parts: the generation of edge features $F^{e}$ and the generation of the learning target for edge features $E^{\mathrm{gt}}$. Specifically, $F_{1}$ and $F_{4}$ are fed into two separate 1 × 1 convolution layers and upsampled. Then, the obtained features are concatenated to generate a new feature. Next, the feature is fed into two consecutive ${CU}_{3}$ and ${CU}_{1}$ layers, and the edge features are output through a sigmoid function for learning the object boundaries, which can be expressed as follows:

(17) $$F_{edge_{t}}=Cat\left(CU_{1}\left(F_{1}\right),CU_{1}\left(F_{4}\right)\right)$$

(18) $$F_{e}=sigmoid(CU_{1}\left(CU_{3}\left(F_{edge_{t}}\right)\right)$$

where $CU_{1}$ refers to a 1 × 1 convolution layer followed by an upsampling operation, $CU_{3}$ refers to a 3 × 3 convolution layer followed by an upsampling operation, and sigmoid(·) denotes the sigmoid activation function. In the generation phase of $E^{\mathrm{gt}}$, we obtain the edge map of the salient object in the final output of each iteration. This edge map guides the ECM in learning the edges of the prediction results. This process can be expressed as:

(19) $$E^{gt}=sobel\left(S^{1}\right)$$

where $S^{1}$ represents the prediction results of the ASPN in each iteration and sobel(·) denotes the edge extraction using the sobel operator.

#### 3.3.4. Progressive Decoder

**Figure 5:**
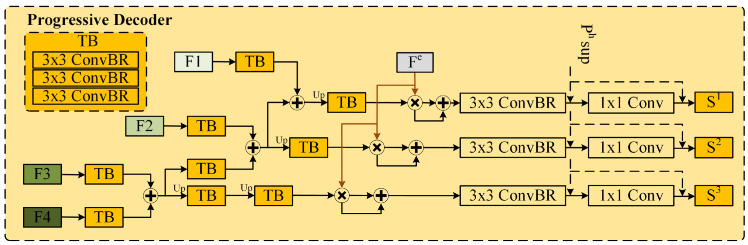
Architecture of progressive decoder.

To obtain the prediction result $S^{o}$, we use a progressive decoder. We first feed $F_{3}$ and $F_{4}$ into the TB layer. The features from the TB layer are then summed element-wise to generate enhanced features. These refined features are subsequently split into two branches: one branch performs upsampling before being fed back into the TB layer, while the other branch combines with $F_{2}$ in the same manner as the previous step. After incorporating $F_{1}$ into the decoding process, the edge features are added by element-wise multiplication during the decoding process, as shown in Figure 5. The decoder equation is as follows:

(20) $$S_{f}=\delta_{↑}\left(TB\left(\delta_{↑}\left(TB\left(F_{i}\right)⊕TB\left(F_{i-1}\right)\right)\right)\right)$$

(21) $$D_{t}=ConBR\left(S_{f}⊕\left(S_{f}⊗Sig\left(F_{e}\right)\right)\right)$$

where $\left\{D_{t}|t=1,2,3\right\}$ represents the intermediate features, ConBR is a 3 × 3 convolution layer followed by BN and ReLU layers, TB consists of three ConBR blocks stacked together, $F_{i}$ denotes the i-th level feature, $\;\delta_{↑}$ represents the upsampling operation, and Sig$\left(\cdot \right)$ is the sigmoid activation function.

Finally, a 1 × 1 convolution layer is used as the projection head for the salient features, outputting the salient object image $S^{o}$, expressed as:

(22) $$S^{o}=Conv_{1}\left(D_{t}\right)$$

Here, $\left\{S^{o}|o=1,2,3\right\}$ is the predicted result and $Conv_{1}\left(\cdot \right)$ represents the convolution operation with a 1 × 1 convolution kernel. We take $S^{1}$ as the final saliency map.

### 3.4. Hybrid Loss Function

Since the pseudo-labels generated by the CPGN provide rich supervisory information and suppress the noise introduced by weak labels, we employ a hybrid loss function that includes pixel-level contrast loss $L_{\mathrm{PCL}}$, enhanced hybrid loss $L_{\mathrm{HEL}}$, and edge loss $L_{E}$ to train the ASPN. $L_{\mathrm{PCL}}$ treats the pseudo-labels and the pixels with the same predictions as positive sample pairs, while the rest are considered negative sample pairs. It trains the ASPN by increasing the similarity of positive pairs and decreasing the similarity of negative pairs. The total loss function is given by the following expression:

(23) $$L=\lambda_{1}\times L_{PCL}+\lambda_{2}\times L_{HEL}+\lambda_{3}\times L_{E}$$

We fix the value of $\lambda_{1}$ to $\lambda_{3}$ in the order of 0.9, 0.1, 1, in all our experiments, where $L_{\mathrm{PCL}}$ is defined as:

(24) $$\begin{array}{c} L_{PCL}=\beta_{1}\times L_{PCL_{1}}\left(D_{1},P^{h}\right)+\\ \beta_{2}\times L_{PCL_{2}}\left(D_{2},P^{h}\right)+\beta_{3}\times L_{PCL_{3}}\left(D_{3},P^{h}\right) \end{array}$$

where $\left\{D_{1},D_{2},\left.D_{3}\right\}\right.\in D_{t}$ is the output before the projection head of the final layer of the progressive decoder. $L_{HEL}$ is defined as:

(25) $$\begin{array}{c} L_{HEL}=\beta_{1}\times L_{HEL_{1}}\left(S^{1},P^{h}\right)+\\ \beta_{2}\times L_{HEL_{2}}\left(S^{2},P^{h}\right)+\beta_{3}\times L_{HEL_{3}}\left(S^{3},P^{h}\right) \end{array}$$

$L_{\mathrm{HEL}}$ is proposed in [40], where $\left\{S^{1},S^{2},\left.S^{3}\right\}\right.\in S^{o}$ is the output after the projection head of the final layer of the progressive decoder. $L_{\mathrm{HEL}}$ optimizes the predicted results from both the edge and region perspectives. We fix the value of $\beta_{1}$ to $\beta_{3}$ in the order of 1, 0.5, 0.5, in all our experiments.

Since the foreground in the edge image contains only a small number of pixels, it may be dominated by the abundance of background pixels. Therefore, we use $L_{\mathrm{Dice}}$ and $\omega L_{\mathrm{BCE}}$ to optimize the edge features. $L_{\mathrm{Dice}}$ is robust to the imbalance between positive and negative samples. It focuses more on the overlapping part of the two sets in the predicted results and pseudo-labels, rather than simply considering the number of elements in each set. This allows $L_{\mathrm{Dice}}$ to better balance the contributions of foreground and background pixels when the pixel distribution is uneven (e.g., when foreground pixels are far fewer than background pixels). $\omega L_{\mathrm{BCE}}$ is adjusted by weighting to balance, and ω is calculated based on the ratio of foreground pixels to background pixels in $E^{\mathrm{gt}}$. We use a combination of $L_{\mathrm{Dice}}$ and $\omega L_{\mathrm{BCE}}$ to optimize the edge features. $L_{E}$ is defined as:

(26) $$L_{E}=L_{Dice}\left(F_{e},E^{gt}\right)+\omega L_{BCE}\left(F_{e},E^{gt}\right)$$

## 4. Experiment and Result Analysis

To maximize the effectiveness of our proposed model, we followed the traditional training setup and performed re-annotation. We selected 1485 images from the NLPR [41] dataset and 700 images from the NJU2K [42] dataset to construct a new cross-modal dataset, CWS. Furthermore, we tested our model on the public datasets LFSD [43], DES [44], SIP [45], DUT-RGBD [46], NLPR [41], NJU2K [42], and STERE [47].

### 4.1. Evaluation Metrics

We evaluate the performance of the models on four golden evaluation metrics, i.e., S-measure, F-measure, E-measure, and mean absolute error (MAE).

### 4.2. Implementation Details

During the construction of the training set, we utilized the CLIP pre-trained ViT-B-16 [48] model for CAM generation. The feature map used to generate the CAM was extracted from the final feature map before the last self-attention layer of ViT. Our model was trained on a single NVIDIA GTX 3090 GPU with 24 GB of memory. The input RGB and depth images were resized to 256 × 256. To avoid overfitting during training, all images were augmented with data augmentation techniques such as random flipping, random cropping, random rotation, and color enhancement. With the backbone network for the RGB stream employed by the Swin-Transformer model pre-trained on ImageNet, we used the AdamW optimizer with $\beta_{s}=\left(0.9,0.999\right)$, $\varepsilon =1\times 10^{-8}$, and a weight decay of $1\times 10^{-4}$ to optimize our model. For the proposed PCL, we set the temperature hyperparameter τ=0.3. The initial learning rate was set to $5\times 10^{-5}$, and we applied a polynomial decay strategy with the formula: $lr=init_{lr}\times \left(1-{\left(curr_{lr}/max_{iter}\right)}^{power}\right)$ where power=0.9. The model was trained for 300 epochs with a batch size of 6.

### 4.3. Performance Comparison with the State of the Art

We compare our proposed method with both weakly supervised and fully supervised models, including MIRV [15], SSSD [32], DENet [17], and SCWS [31], which are based on scribble supervision; PSOD [34], which is based on point supervision; and MSOD [49], JSM [38], and MSW [35], which are based on multiple weak labels. For pixel-level supervision, we compare CPNet [50], HFIL [51], TPCL [28], CATNet [11], and C2DFNet [52]. To ensure a fair comparison, we either used the salient object maps provided by the authors or ran the models released by the authors to predict on the RGB-D datasets. For models designed for RGB images, we only input the RGB images from the RGB-D datasets for comparison.

#### 4.3.1. Quantitative Evaluation

Our method was compared against state-of-the-art single-label weakly supervised, multiple-label weakly supervised, and fully supervised SOD models for RGB-D images. We evaluated the performance across all datasets listed in Table 1 using the F-measure ($F_{\beta }↑$), S-measure ($S_{\alpha }↑$), E-measure ($E_{m}↑$), and MAE (M↓). Among the compared methods, MSOD, JSM, and MSW employ two or more weak labels as supervision sources, similar to our approach, while MIRV, Denet, SCWS, and POSD rely on a single supervision source. CPNet, HFIL, TPCL, CATNet, and C2DFNet, on the other hand, are fully supervised models using pixel-level labels.

As shown by the results, our model achieves state-of-the-art performance on datasets with rich filamentary structures (SIP) and those with complex scenes (DUT-RGBD). Although our method lags behind on the DES dataset, further investigation revealed that this is due to the dataset’s limited size (only 135 images) and high homogeneity, with simple structures that diminish the advantages of our method in handling complex structural information.

#### 4.3.2. Qualitative Evaluation

**Figure 6:**
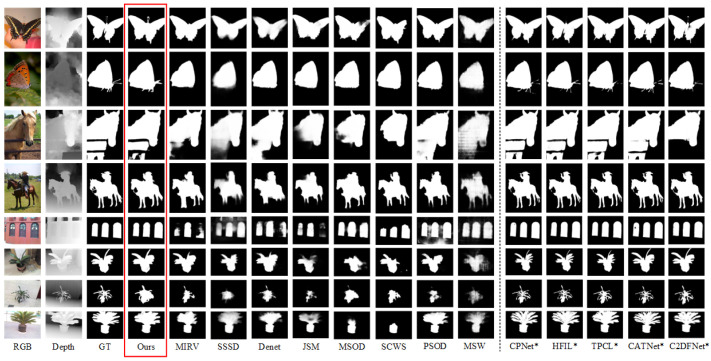
The results of our model are compared with those of previous models. Our method is indicated in the red box. Columns 4 to 8 are weakly supervised models for RGB-D images, columns 9 to 12 are weakly supervised models for RGB images, and columns 13 to 17 are fully supervised models for RGB-D images. We denote full-supervision methods by adding an asterisk “*” after the method names.

To further demonstrate the effectiveness of our method, Figure 6 shows a comparison with state-of-the-art methods in various scenes. It can be seen that compared to single weakly supervised models (such as MIRV, SSSD, DENet, SCWS, and PSOD), our model exhibits a stronger ability to localize salient objects and segment complete regions. When compared to multiple weakly supervised models (such as MSOD, JSM, and MSW), our model produces smoother salient object edges. Notably, our model shows an overwhelming advantage in representing fine filamentary structures (e.g., rows 1, 2, 4, 6, and 7), even demonstrating competitive performance when compared to fully supervised tasks. For occluded objects (e.g., row 3), our model is also able to accurately identify and segment salient objects, exhibiting a superior understanding of complex spatial structures—surpassing even fully supervised models like CATNet and C2DFNet, which aligns with the original intent of the SOD task for RGB-D images. Furthermore, our model excels in recognizing multiple objects (e.g., row 5), where it accurately segments the structures of multiple targets without introducing background noise. Overall, our model surpasses the limitations of weak labels in object completeness recognition and remains competitive even among fully supervised models.

#### 4.3.3. Complexity Comparisons

We calculate the FLOPs and parameters for weakly supervised methods based on RGB-D images, and the results are shown in Table 2 below. The FLOPs and parameters of our model are relatively high. However, compared with other methods, our method achieves higher detection performance for filamentary structures. Additionally, it is worth noting that the primary computational cost of our model lies in the Swin-Transformer backbone, which accounts for 87 M parameters.

### 4.4. Ablation Studies

We conducted various ablation studies to validate the effectiveness of each key component of the model on the RGB-D dataset. This includes an examination of the effectiveness of weakly supervised pseudo-labels in relation to the model architecture. We quantitatively present the benefits of each component in a table. Our model achieved the best performance when all components were utilized.

We selected four datasets as the test datasets for the ablation experiments. The NJU2K and NLPR are two representative datasets for the SOD task on RGB-D images. The depth maps have better quality in DUT-RGBD, while DES represents a case where our method performs relatively poorly. The performance on these four datasets is sufficient to represent the effectiveness of our method.

#### 4.4.1. Effectiveness of Cross-Modal Weak Labels

**Figure 7:**
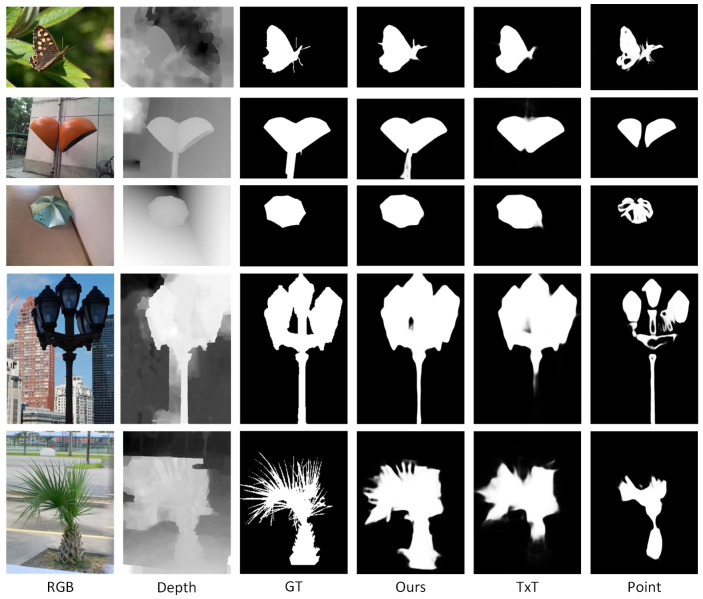
Ablation study results comparison of cross-modal weak labels.

As shown in Table 3, we disassembled our cross-modal weak labels to train the model with the pseudo-labels formed by the text labels and the point labels, respectively. We found that the performance of the model was significantly reduced when only single weak labels were used, which was sufficient to prove that a single source was not enough to meet the requirements of SOD tasks for RGB-D images. Figure 7 shows the comparison of prediction results using cross-modal weak labels and using single-modal weak labels. It can be seen from the figure that when we only use text labels, the background is noisy, and when only point labels are used, the predicted results are incomplete. These are consistent with the defects of single-modal weak labels, respectively, while the prediction results obtained by the combination of cross-modal weak labels proposed by us have abundant filamentous structures and a clean background.

#### 4.4.2. Effectiveness of Asymmetric Encoder and ECM

**Figure 8:**
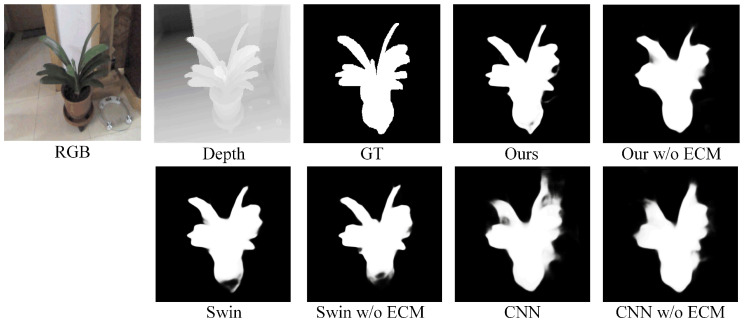
The effectiveness of the asymmetric encoder and the ECM. “Swin” refers to the symmetric encoder structure based on the Swin-Transformer, and “CNN” refers to the symmetric encoder structure based on a CNN.

We added two sets of comparative experiments using symmetric encoders to verify the effectiveness of the asymmetric architecture. Moreover, we validated the effectiveness of the ECM in each set of experiments. The qualitative evaluation is shown in Figure 8, where it can be clearly observed that our asymmetric structure better captures the filamentary structure of the salient object. The ECM also sharpens the edges of the salient region. The experimental results are shown in Table 4. From the quantitative results, we observed that the asymmetric structures based on the Swin-Transformer and CNN encoders achieved better performance in the experiments without the ECM. After incorporating the ECM into the network, the performance improved significantly, which strongly demonstrates the effectiveness of the proposed asymmetric encoder combined with the ECM.

## 5. Discussion

Weakly supervised salient object detection holds great potential for applications in medical imaging, defect detection, and autonomous driving. The reduced annotation cost and accurate localization of salient objects provide a solid technical foundation for practical applications. The introduction of depth information can enhance the ability of the predictive model to recognize objects in low-contrast or complex background scenarios. In this study, we propose a cross-modal weak supervision framework that integrates text and point labels to provide both semantic and pixel-level guidance. This approach helps the model to learn a more complete object contour, especially in capturing filamentary structures. It also introduces depth information as guidance to overcome adverse environmental factors and to clearly delineate the geometric information of the object. These advantages contribute to the competitiveness of our approach in real-world applications. Our method significantly improves the detection of complete salient objects by aggregating multi-modal and multi-dimensional information. In future work, how to use a small amount of supervision information to complete the detection of complex structures is expected to become a development direction for weakly supervised salient object detection, and this is also the main issue we need to explore.

## 6. Conclusions

We propose a high-performance cross-modal weakly supervised salient object detection (SOD) framework for RGB-D images that consists of two main components: pseudo-label generation and salient object detection. Since the issue of weak labels is insufficient supervision, we propose the CPGN, which can overcome the limitations of different weak labels by combining the advantages of text labels and point labels, thus generating high-quality pseudo-labels to train the ASPN and providing sufficient and accurate saliency information. Additionally, considering the attribute differences between RGB and depth images, we utilize an asymmetric encoder structure, where RGB-D image features are extracted using a Swin-Transformer and CNN-based encoders, respectively. To further enhance the model’s perception of edge information, we propose the ECM, which constrains the edges of salient objects through multi-scale feature integration. Based on experimental results from seven datasets, our proposed method outperforms other weakly supervised approaches and demonstrates competitive performance even when compared to fully supervised methods in fine filamentary structure segmentation.

## Figures and Tables

**Table 1:** The benchmark test results of the SOD model. ↑ and ↓ denote that the larger and the smaller are better. Among them, sup. represents the supervision method used by the model, which is divided into weak supervision and full supervision. We denote full-supervision methods by adding an asterisk “*” after the method names. We separately highlight the best performance indicators of weak supervision and full supervision in bold.

	Sup.	Weakly Sup.									Fully Sup.				
		Multiple Weak Labels Sup.				Single Weak Label Sup.									
DATASET	Mtric	Our	MSOD	JSM	MSW	MIRV	SSSD	Denet	SCWS	PSOD	CPNet *	HFIL *	TPCL *	CATNet *	C2DFNet *
			[49]	[38]	[35]	[15]	[32]	[17]	[31]	[34]	[50]	[51]	[28]	[11]	[52]
DUT-RGBD [46]	$S_{\alpha }↑$	**0.887**	0.828	0.791	0.825	0.876	0.877	0.845	0.857	0.887	0.951	0.950	0.935	**0.953**	0.930
	$F_{\beta }↑$	**0.881**	0.824	0.802	0.777	**0.881**	0.876	0.830	0.865	0.885	**0.956**	0.953	0.940	0.951	0.934
	$E_{\xi }↑$	**0.937**	0.885	0.870	0.877	0.919	0.922	0.894	0.902	**0.937**	**0.976**	0.975	0.966	0.971	0.958
	M↓	**0.049**	0.078	0.093	0.104	0.054	0.067	0.071	0.060	0.054	**0.019**	**0.019**	0.024	0.020	0.025
SIP [45]	$S_{\alpha }↑$	0.870	0.735	0.707	0.781	**0.876**	0.876	0.852	0.833	0.870	0.907	0.908	0.900	**0.913**	0.872
	$F_{\beta }↑$	**0.893**	0.708	0.691	0.709	0.872	0.872	0.840	0.830	0.865	**0.925**	0.923	0.914	0.918	0.867
	$E_{\xi }↑$	**0.926**	0.812	0.787	0.836	0.925	0.925	0.904	0.900	0.926	0.944	**0.946**	0.941	0.944	0.915
	M↓	**0.049**	0.125	0.141	0.127	**0.049**	0.060	0.063	0.065	0.050	0.035	**0.034**	0.037	**0.034**	0.054
NJU2K [42]	$S_{\alpha }↑$	0.891	0.802	0.723	0.784	0.890	**0.902**	0.883	0.853	0.884	0.934	0.936	0.925	**0.937**	0.912
	$F_{\beta }↑$	0.891	0.805	0.744	0.740	0.888	**0.904**	0.870	0.870	0.882	**0.936**	**0.936**	0.924	0.929	0.912
	$E_{\xi }↑$	0.939	0.856	0.793	0.840	0.929	**0.950**	0.915	0.900	0.921	**0.960**	0.959	0.955	0.933	0.919
	M↓	**0.040**	0.091	0.129	0.119	0.045	0.048	0.050	0.059	0.048	**0.024**	0.025	0.028	0.025	0.038
DES [44]	$S_{\alpha }↑$	0.909	0.859	0.826	0.835	**0.928**	0.918	0.900	0.854	0.895	**0.949**	0.946	0.935	0.945	0.914
	$F_{\beta }↑$	0.878	0.831	0.827	0.777	**0.927**	0.909	0.890	0.877	0.897	**0.937**	0.935	0.923	0.914	0.896
	$E_{\xi }↑$	0.957	0.927	0.890	0.890	**0.972**	0.967	0.957	0.886	0.944	**0.984**	0.980	0.971	0.979	0.955
	M↓	0.022	0.042	0.056	0.065	**0.018**	0.028	0.028	0.037	0.028	**0.012**	0.013	0.016	0.016	0.021
NLPR [41]	$S_{\alpha }↑$	0.903	0.844	0.810	0.825	**0.913**	0.899	0.902	0.867	0.885	0.940	**0.942**	0.935	0.939	0.928
	$F_{\beta }↑$	0.866	0.792	0.789	0.728	**0.902**	0.884	0.874	0.842	0.857	**0.929**	0.927	0.920	0.916	0.899
	$E_{\xi }↑$	0.948	0.879	0.890	0.840	**0.954**	0.947	0.943	0.913	0.921	**0.972**	**0.972**	0.968	0.968	0.958
	M↓	**0.023**	0.050	0.058	0.075	0.025	0.035	0.031	0.039	0.038	**0.016**	**0.016**	0.017	0.018	0.021
LFSD [43]	$S_{\alpha }↑$	**0.862**	0.803	0.766	0.808	0.854	0.835	0.832	0.806	0.848	0.892	0.885	0.885	**0.898**	0.863
	$F_{\beta }↑$	**0.874**	0.811	0.799	0.780	0.861	0.837	0.827	0.825	0.845	0.897	0.887	0.883	**0.900**	0.863
	$E_{\xi }↑$	**0.905**	0.859	0.823	0.840	0.901	0.879	0.868	0.849	0.903	0.925	0.917	0.918	**0.933**	0.883
	M↓	**0.069**	0.102	0.128	0.129	0.070	0.095	0.089	0.098	0.087	0.049	0.058	0.058	**0.040**	0.065
STERE [47]	$S_{\alpha }↑$	0.895	0.842	0.858	0.838	0.890	0.881	0.879	0.876	**0.899**	0.920	0.922	0.916	**0.925**	0.902
	$F_{\beta }↑$	0.877	0.831	0.796	0.785	0.880	0.870	0.855	0.885	**0.892**	0.909	**0.910**	0.902	0.902	0.892
	$E_{\xi }↑$	**0.943**	0.892	0.858	0.880	0.936	0.929	0.921	0.930	0.940	**0.954**	0.953	0.951	0.935	0.927
	M↓	**0.035**	0.065	0.092	0.089	0.041	0.059	0.051	0.044	0.039	**0.029**	0.030	0.031	0.030	0.038

**Table 2:** Comparison of FLOPs and parameters among different methods.

Method	FLOPs (G)	Params (M)
Ours	274.19	112.66
SSSD [32]	44.28	130.78
JSM [38]	17.94	47.85
DeNet [17]	179.71	18.57
MIRV [15]	37.14	63.58

**Table 3:** Effectiveness of our cross-modal weak labels strategy.

	NJU2K				NLPR			
Sup.	$S_{\alpha }↑$	$F_{\beta }↑$	$E_{m}↑$	M↓	$S_{\alpha }↑$	$F_{\beta }↑$	$E_{m}↑$	M↓
TXT	0.814	0.807	0.875	0.081	0.875	0.886	0.921	0.037
Point	0.672	0.747	0.744	0.134	0.724	0.785	0.804	0.067
Ours	**0.891**	**0.891**	**0.939**	**0.040**	**0.903**	**0.866**	**0.948**	**0.023**
	DUT-RGBD				DES			
Sup.	$S_{\alpha }↑$	$F_{\beta }↑$	$E_{m}↑$	M↓	$S_{\alpha }↑$	$F_{\beta }↑$	$E_{m}↑$	M↓
TXT	0.818	0.811	0.889	0.088	0.879	0.835	0.920	0.038
Point	0.705	0.793	0.777	0.110	0.684	0.749	0.750	0.072
Ours	**0.887**	**0.881**	**0.937**	**0.049**	**0.909**	**0.878**	**0.957**	**0.022**

The best results are highlighted in bold. ↑ and ↓ denote that the larger and the smaller are better.

**Table 4:** The effectiveness of the asymmetric encoder and the ECM. “w/o” refers to “without”.

	NJU2K				NLPR				DUT-RGBD				DES			
**Model**	$S_{\alpha }↑$	$F_{\beta }↑$	$E_{m}↑$	M↓	$S_{\alpha }↑$	$F_{\beta }↑$	$E_{m}↑$	M↓	$S_{\alpha }↑$	$F_{\beta }↑$	$E_{m}↑$	M↓	$S_{\alpha }↑$	$F_{\beta }↑$	$E_{m}↑$	M↓
CNN + CNN w/o ECM	0.807	0.821	0.846	0.097	0.796	0.814	0.833	0.069	0.783	0.794	0.835	0.110	0.832	0.748	0.870	0.054
CNN + CNN	0.832	0.841	0.866	0.089	0.828	0.825	0.864	0.060	0.791	0.812	0.844	0.098	0.891	0.787	0.891	0.045
Swin-Transformer + Swin-Transformer w/o ECM	0.847	0.856	0.886	0.068	0.869	0.830	0.903	0.043	0.857	0.830	0.903	0.065	0.869	0.841	0.914	0.033
Swin-Transformer + Swin-Transformer	0.858	0.882	0.904	0.059	0.879	0.833	0.919	0.033	0.869	0.845	0.928	0.054	0.888	0.848	0.936	0.029
Ours w/o ECM	0.853	0.860	0.896	0.063	0.873	0.820	0.914	0.036	0.86	0.849	0.919	0.066	0.879	0.835	0.925	0.032
Ours	**0.891**	**0.891**	**0.939**	**0.040**	**0.903**	**0.866**	**0.948**	**0.023**	**0.887**	**0.881**	**0.937**	**0.049**	**0.909**	**0.878**	**0.957**	**0.022**

The best results are highlighted in bold. ↑ and ↓ denote that the larger and the smaller are better.

## Data Availability

Data are contained within the article.
